# *Fusarium chlamydosporum*, causing wilt disease of chili (*Capsicum annum* L.) and brinjal (*Solanum melongena* L.) in Northern Himalayas: a first report

**DOI:** 10.1038/s41598-022-23259-w

**Published:** 2022-11-27

**Authors:** Tasmeen J. Parihar, Mohd. Yaseen Sofi, Rovidha S. Rasool, Sehla Khursheed, Zahoor A. Bhat, Khursheed Hussain, Bhagyashree Dhekale, Sajad Majeed Zargar, Asma S. Hakak, Mehraj D. Shah, F. A. Nehvi, M. Ashraf Bhat, M. N. Khan, Khalid Z. Masoodi

**Affiliations:** 1 Transcriptomics Laboratory (K-Lab), Division of Plant Biotechnology, Sher-e-Kashmir University of Agricultural Sciences and Technology of Kashmir (SKUAST-Kashmir), Shalimar, Srinagar, Jammu and Kashmir 190025 India; 2 IDP-NAHEP, Sher-e-Kashmir University of Agricultural Sciences and Technology of Kashmir, Shalimar, Srinagar, Jammu and Kashmir 190025 India; 3 Division of Plant Pathology, Sher-e-Kashmir University of Agricultural Sciences and Technology of Kashmir, Shalimar, Srinagar, Jammu and Kashmir 190025 India; 4 Division of Vegetable Science, Sher-e-Kashmir University of Agricultural Sciences and Technology of Kashmir, Shalimar, Srinagar, Jammu and Kashmir 190025 India; 5 Division of Agricultural Statistics, Sher-e-Kashmir University of Agricultural Sciences and Technology of Kashmir, Shalimar, Srinagar, Jammu and Kashmir 190025 India; 6 Proteomics Laboratory, Division of Plant Biotechnology, Sher-e-Kashmir University of Agricultural Sciences and Technology of Kashmir, Shalimar, Srinagar, Jammu and Kashmir 190025 India; 7 College of Temperate Sericulture, SKUAST-Kashmir, Mirgund, Jammu and Kashmir 193121 India

**Keywords:** Plant biotechnology, Microbiology, Molecular biology, Plant sciences

## Abstract

Chili (*Capsicum annuum* L.) and brinjal (*Solanum melongena* L.) are the most widely grown solanaceous crops in the world. However, their production has reduced over several years due to the attack of various fungal and bacterial pathogens and various abiotic factors. Still, the major constrain in their production are pathogens with fungal etiology, especially the fungal wilt of solanaceous crops. *Fusarium oxysporum* and *Fusarium solani* have been previously identified as the pathogens causing wilt disease in chili and brinjal. Recently, a new fungal pathogen *F. equiseti* has been reported as the causal agent of wilt disease infecting chili. The current study focused on identifying fungal pathogens associated with the wilted plants of chili and brinjal, collected from different parts of the Himalayan region of Kashmir valley, through morpho-cultural and molecular characterization. DNA extraction, PCR amplification, and sequencing were performed on various isolates. DNA barcoding using the internal transcribed spacer region (ITS) was used to identify the pathogen followed by the pathogenicity test. Further confirmation of the pathogen was done by sequencing of transcription elongation factor (TEF) and Calmodulin (CAL2). In current study *Fusarium chlamydosporum* has been reported as the wilt causing pathogen of chili and brinjal for the first time in Kashmir Himalayas.

## Introduction

The most widely grown and commercially important vegetable crops in India are chili (*Capsicum annuum* L.) and brinjal (*Solanum melongena* L.).India is the primary producer, exporter, and consumer of chili globally, while it ranks second in world brinjal production after China^1^. Both these crops belong to the solanaceous family and are considered the vital source of nutrients, thus helping in combating various human health diseases and disorders. These crops are prone to multiple diseases, and the most devastating is the wilt in terms of incidence and yield loss^2^. In India, the wilt of chili and brinjal has emerged as a severe threat, with disease incidence of 2–85%^3^. The two critical fungal species i.e., *Fusarium oxysporum* and *Fusarium solani* are involved with chili wilt in India, whereas, *Fusarium moniliforme and Fusarium pallidoroseum* are the other two species found in some parts of India^3,4^. Recently, *F. equiseti* has been reported from Kashmir valley as the causal agent of chili wilt^5^. Global yield losses owing to the disease are estimated to range between 10 and 80%^6^. Apart from chili and brinjal, wilt affects a wide variety of crops such as tomato, tobacco, legumes, cucurbits, etc. The characteristic symptoms of wilt disease are slight yellowing of the leaves that wither and defoliate prematurely. Browning of the vascular tissues, stunting, and necrosis are the other symptoms^7,8^. Reports suggest that *Fusarium palfidoroseum Sacc*. is the primary causal agent of wilt in Kashmir valley*.* The losses due to this disease is reported to be 30–40% annually^9^.

The identification of the *Fusarium* spp. is conventionally based on morphological characteristics such as shape, size, and color of micro and macroconidia besides colony characteristics^5^. Although the primary identification of the pathogen is based on the colony and morphological characters, the information collected by nucleotide sequencing from conserved gene sections, such as internal transcribed spacer and translation elongation factor 1-alpha, is the most reliable method of identification. Recently *F. equiseti* has been reported as the cause of chili wilt through DNA barcoding^5^.

Various approaches, such as direct sequencing of the ITS region and restriction fragment patterns from PCR-generated ITS sequences, are viewed as effective methods for identifying fungi at the species level. These methods have been utilized in the molecular identification of a wide range of pathogenic fungi^10^. Besides ITS, transcription elongation factor (TEF) is also used to identify the associated pathogens. In the current study, an attempt was made to identify the pathogens associated with chili and brinjal wilt, based on morphological and molecular characterization using different DNA Barcodes viz., internal transcribed spacer, translation elongation factor 1-alpha and Calmodulin (CAL2) ^11^.

## Material and methods

### Survey for disease incidence

A survey of three districts (Srinagar, Baramulla, and Anantnag) in Kashmir Division of Northern Himalayan region of India was carried out during July–August 2019 through 2021 to assess the status of wilt disease of chili and brinjal. Field studies and plant material collections were conducted in accordance with local legislation, and appropriate permissions were obtained as and when required. In the present study, SKUAST-K has various KVKs in all districts of J&K that directly work with local farmer populations under various schemes. There is a mutual agreement between the University and the Department of Agriculture, J&K for sharing the material for research. Three villages were selected from each district, and three fields were chosen randomly in each village. One square meter area (1 m^2^) from each field involving approximately 20–25 plants was selected randomly to assess the disease incidence. The percent disease incidence was calculated using the following formula:

$${\text{Percent disease incidence = }}\frac{{\text{Total number of diseased plants}}}{{\text{Total number of examined plants }}} \, { \times }{1}00$$

### Isolation, identification and pathogenicity test of the pathogen isolate

The pathogen isolates were isolated on PDA medium using the tissue bit method from infected samples. The cultures were purified by single spore technique and maintained on PDA slants at 25 ± 1 °C^12,13^. The pathogen was identified based on morphological (shape, size, and color of micro and macroconidia and septation) and cultural characters (colony color) of the isolated cultures on the PDA and SNA media. DNA barcoding was further used to authenticate morphological-based identification. The pathogenicity test of the isolated fungus was performed on potted chili (cv. Shalimar Long) and brinjal (cv. Local Long) plants using the rhizosphere inoculation technique^14^ to prove Koch’s postulates. Sand maize meal medium (potting mixture) was prepared by placing 90 g of sand and 10 g of maize meal in 250 ml of Erlenmeyer flask that contained 40 ml of distilled water was autoclaved at 1.05 kg cm^−2^ pressure for half an hour. Spore suspension of the pathogen was prepared from the already isolated and purified pathogen that was grown earlier on PDA (Potato dextrose agar) medium and inoculated in the sterilized Sand maize meal mixture (potting mixture) in ratio of (2:1) and spore suspension @ 100 µl per pot was mixed with upper layer of soil and continues shaking of this potting mixture inoculated with pathogen was done to obtain uniform growth of the pathogen in potting mixture that was allowed to grow for 7 days to infest soil^5,15,16^ (Supplementary Table 1). Plants of chili and brinjal were sown in this infected potting mixture and grown for 3 weeks to provide conducive conditions for the pathogen to infect the host plants. Finally, the plants were observed for appearance of symptoms.

### Morpho-cultural characterization

Cultural characterization was carried out based on colony color and status of the mycelium that were grown on PDA medium. For morphological studies of the fungus, the wet mounts in lactophenol and cotton blue of 10 days old culture were examined under the microscope, and the observations with respect to color, width, and septation of hyphae, as well as the shape, size, septation, and color of conidia, conidiophore, and chlamydospore were recorded.

### DNA extraction and PCR amplification

DNA was isolated using a 400 μl SDS extraction buffer (200 mM Tris HCL pH 8.5, 250 mM NaCl, 25 mM EDTA, and 0.5% SDS)^17^. Primers were designed manually using Oligocalc, ClustalW (http://www.ebi.ac.uk/Tools/msa/clustalo/) and Blast (http://blast.ncbi.nlm.nih.gov/Blast.cgi) online software. ITS1F2/ITS4R2 and TEF Fu3 F/TEF Fu3 R CAL2-28F/CAL2-RD were used for PCR amplification (Tables 1, 2 and, 3).

**Table 1:** Primers used for amplification of ITS regions.

S. No	Primer name	Primer sequence	Size of amplicon	Tm °C
1	K-Lab-FusOxy-ITS1F2 Primer	5′CCTGCGGAGGATCATTA 3′	~ 500 bp	63.7
2	K-Lab-FusOxy-ITS4R2 Primer	5′TCCTCCGCTTATTGAT3′		53.6

**Table 2:** Primers used for amplification of TEF region.

S. No	Primer name	Primer sequence	Size of amplicon	Tm °C
1	K-Lab-TEF-Fu3-F	5′GGTATCGACAAGCGAACCAT3′	~ 500 bp	63.8
2	K-Lab-TEF-Fu3-R	5′TAGTAGCGGGAGTCTCGAA3		63.8

**Table 3:** Primers used for amplification of CAL2 to identify the species.

S. No	Primer name	Primer sequence	Size of amplicon	Tm °C
1	K-Lab CAL2-28F-F	5′GAGTTCAAGGAGGCCTTCTCCC3′	~ 600 bp	66.3
2	K-Lab CAL2-RD-R	5′TGRTCNGCCTCDCGGATCATCTC3′		74.7

### Sequencing and DNA barcoding

PCR products amplified using ITS1F2/ITS4R2 and TEF Fu3 F/TEF Fu3 R were outsourced for sequencing to Bionivid Technology, Bangalore, India. The genetic variability among collective samples was ascertained through DNA barcoding of ITS1 and ITS2. Dendrogram was constructed using MEGA 6.0 software. The results were reconfirmed using CAL2-28F/CAL2-RD primers for identification and were outsourced for sequencing to Biokart India Pvt. Ltd.

## Results

### Morpho-cultural identification of pathogen isolates

**Figure 1:**
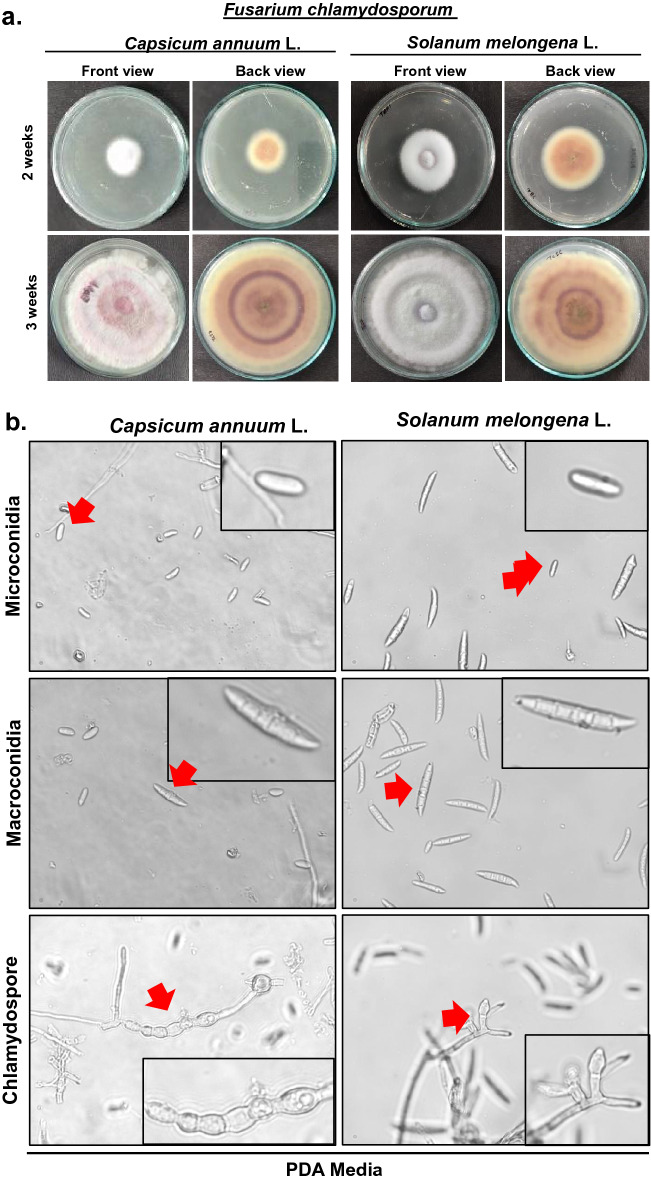

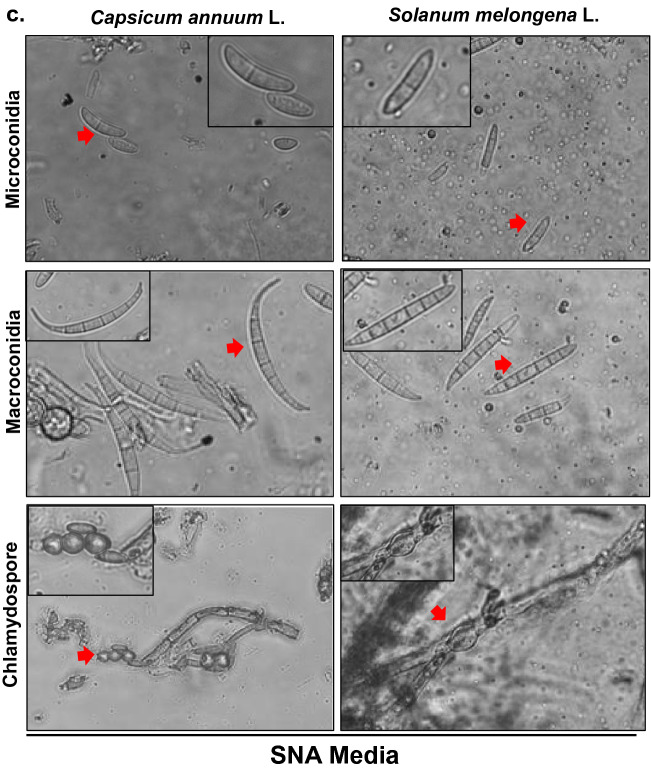
**(a)** Morpho-cultural characteristics of *Fusarium clamydosporum* isolated from wilted plants of chili *Capsicum annuum* L. and brinjal *Solanum melongena *L. showing 2 and 3 weeks old cultures front and back side. (**b)** Microconidia, Macrocondia and chlamydospore of *Fusarium clamydosporum* isolated from wilted plants of chili (*Capsicum annuum *L*.*) and brinjal (*Solanum melongena *L.). (**c)** Microconidia, Macrocondia and chlamydospore of *Fusarium clamydosporum* in SNA medium.

The colonies initially appeared white (Fig. 1a), which progressively became purple at the agar base, which showed 90 mm of growth after incubation of 15 days at 25 ± 1 °C. Mycelium was smooth, branched, cylindrical, septate, and 3.28–4.82 µm wide. The conidiophores were cylindrical, short, septate, and measured 72.50–106.30 × 3.00–4.50 µm in size (Table 4). Microconidia were spindle-shaped, hyaline with 0–3 septa, measuring 6.0–26.0 × 2.0–4.0 µm in size while as macroconidia were sickle-shaped, hyaline with 3–5 septa and measured 30.0–38.0 × 3.0–4.5 µm in size (Fig. 1b and Suppl Fig. 2a, b). The chlamydospores were intercalary, rough walled, hyaline, and measured 5.80–9.01 µm in diameter (Fig. 1b and Suppl Fig. 2c). SNA media being low nutrient medium than PDA helped to obtain more prominent microconidia, macroconidia and, clamadospores (Fig. 1c). Based on these characteristics and their comparison with the authentic description given by Booth (1971)^18,19^, the fungus associated with chili and brinjal was identified as *Fusarium chlamydosporum*.

**Table 4:** Morpho-cultural characteristics of *Fusarium chlamydosporum* causing chili and brinjal wilt.

Fungal propagule	Shape	Color	Size	Septation
Colony	Branched monophialids, cylindrical mycelium	Initially white, turning light pink colored at agar base	90 mm dia. in 15 days	Septate
Hyphae	Smooth and branched	Hyaline	3.28–4.82 µm in width	Septate
Microconidia	Spindle-shaped	Hyaline	6–26 × 2–4 µm	0–3 Septate
Macroconidia	Sickle shaped	Hyaline	30–38 × 3–4.5 µm	3–5 Septate
Conidiophores	Cylindrical, short, and branched	Hyaline	72.50–106.30 × 3.00–4.50 µm	Septate
Chlamydospores	Spherical, smooth, and rough walled, single or in chains	Hyaline	5.81–9.01 µm in dia.	

### Pathogenicity test

**Figure 2:**
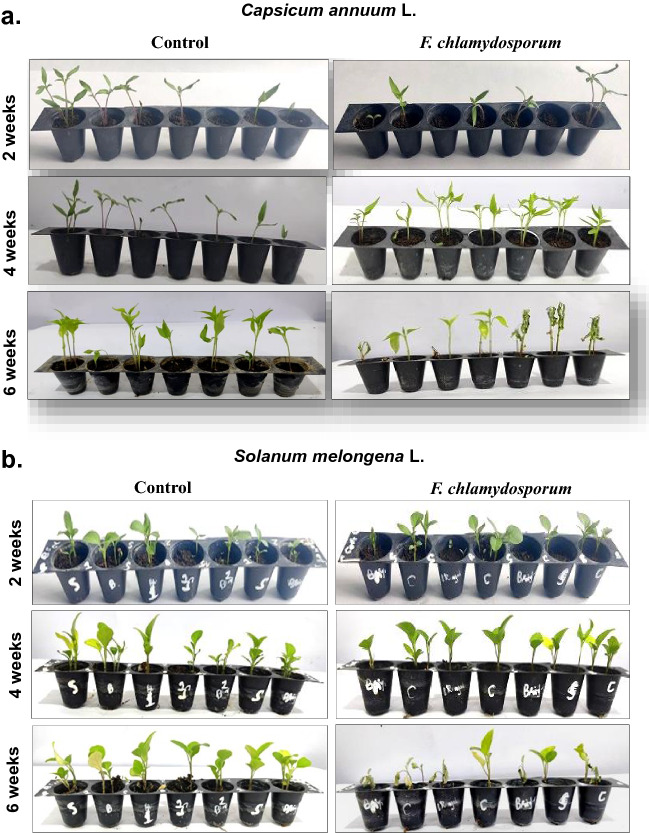
**(a)** Pathogenicity test of isolated *Fusarium clamydosporum* on potted chili *Capsicum annuum* L. plants at 2, 4 and 6 weeks after sowing control plants were treated with distilled water. (**b)** Pathogenicity test of isolated *Fusarium clamydosporum* on potted brinjal *Solanum melongena *L. plants at 2, 4, and 6 weeks after sowing. Control plants were treated with distilled water.

**Figure 3:**
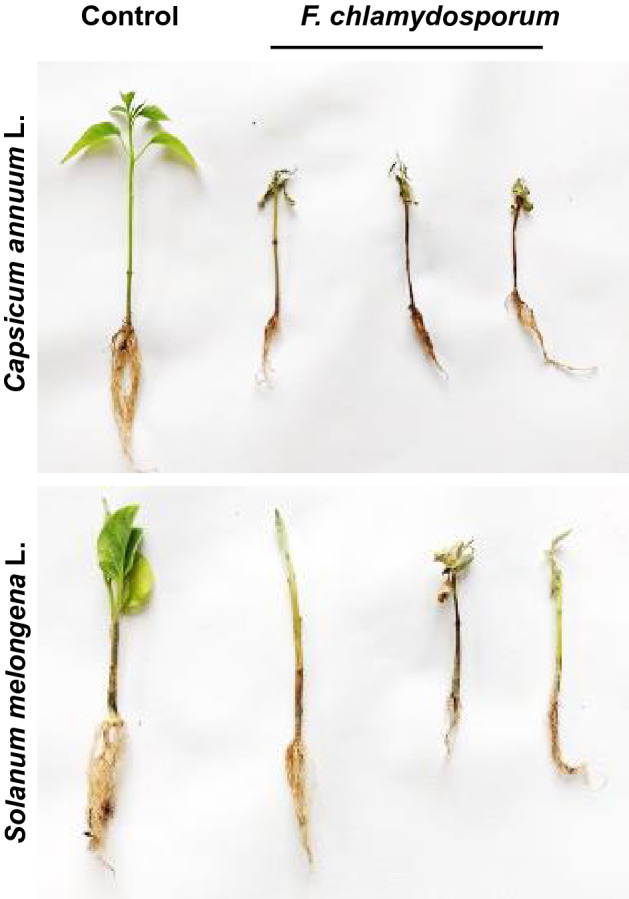
Symptomatological development of wilt diseases of a. chili. (*Capsicum annuum *L.)  and brinjal. (*Solanum melongena* L.) Control depicts uninfected plants and *F. clamydosporum*-treated with pathogen shows wilted plants.

The pathogenicity of isolates was carried out on potted chili (cv. Shalimar Long) and brinjal (cv. Local Long) plants using rhizosphere inoculation technique (Fig. 2 a,b). The incubation period for *F. chlamydosporum* was 19–20 days in brinjal and chili hosts respectively. The initial symptoms appeared as pale green to yellow discoloration of leaves, which later started to droop and finally resulted in the death of the entire plant. Vascular bundles, when exposed, showed brownish discoloration. *F. chlamydosporum* was re-isolated from the inoculated seedlings and was found to be the same pathogen when compared with their original isolates and resembled the original inoculated pathogen in morphological, cultural, and pathogenic characteristics, proving Koch’s postulates (Fig. 3). The cultures were further validated at the molecular level by sequencing of TEF Locus and CAL2 gene.

### DNA extraction and PCR amplification

**Figure 4:**
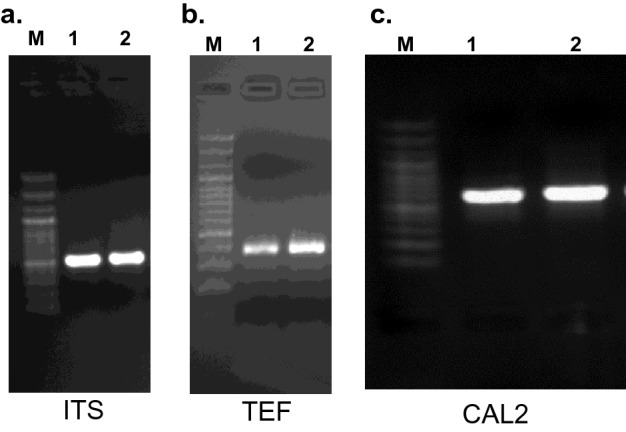
**(a)** PCR product amplified from ITS region of *Fusarium clamydosporum*. M-100 bp ladder, Lane 1—pathogen isolated from chili, Lane 2—pathogen isolated from brinjal. (**b)** PCR product amplified from TEF region of *Fusarium clamydosporum*. M-100 bp ladder, Lane 1—pathogen isolated from chili, Lane 2—pathogen isolated from brinjal. (**c)** PCR product amplified from CAL2 of *Fusarium clamydosporum* M-100 bp ladder, Lane 1—pathogen isolated from chili, Lane 2—pathogen isolated from Brinjal.

**Figure 5:**
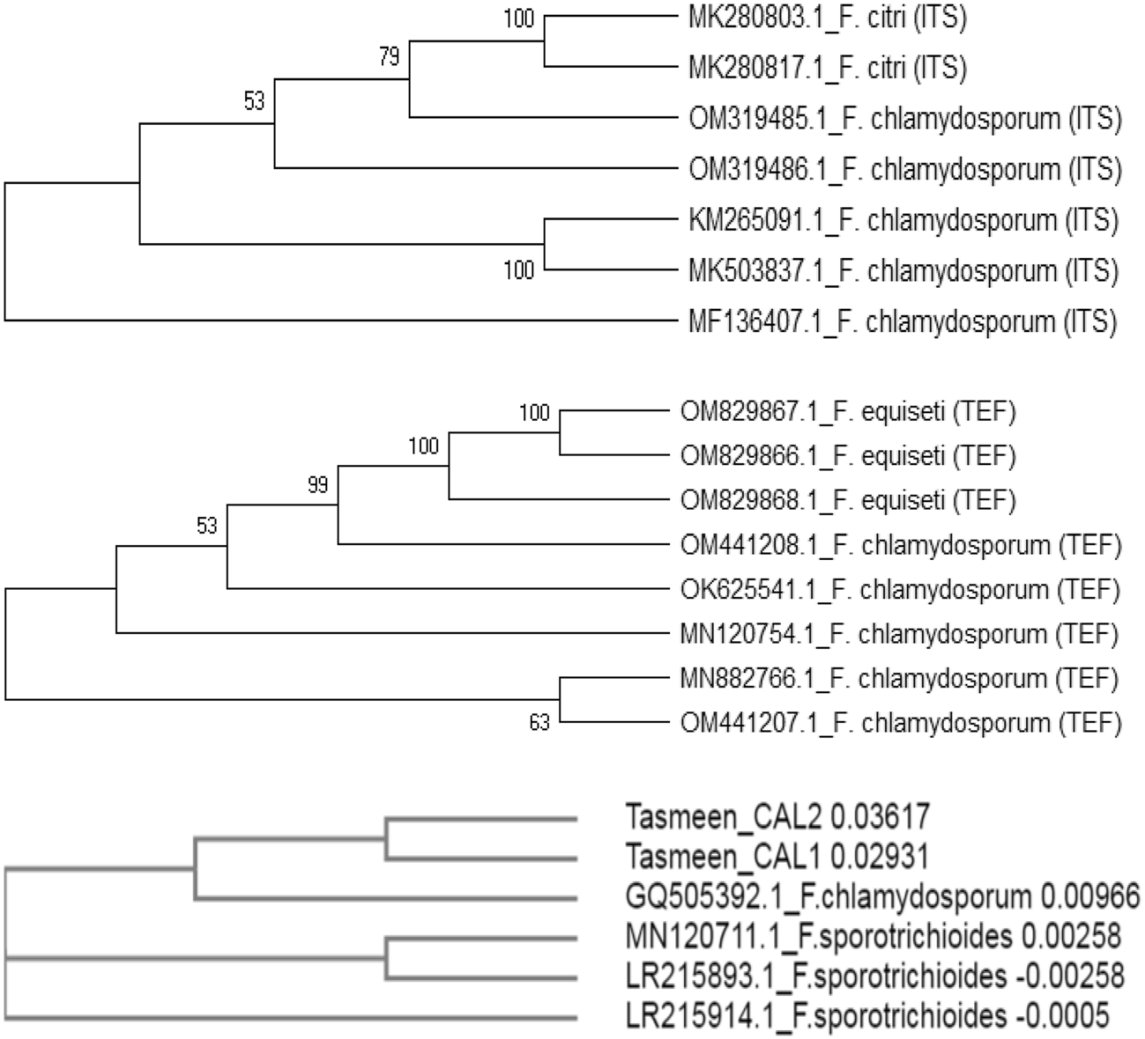
Dendrogram generated by ITS, TEF and CAL2 sequence data predicting similarity and evolutionary relationship between the two isolates from chili and brinjal.

The genomic DNA was extracted from fungal cultures and loaded on 0.7% agarose gel that was run for 30 min. The DNA extracted from different isolates resulted in single high molecular weight intact bands, indicating a good quality DNA. PCR amplification under standard conditions was carried out, and the amplified products were run on 1% agarose gel. PCR showed the amplified product size of 500 bp of ITS1 and ITS2 region (Fig. 4a and Suppl Fig. 1a), PCR product of TEF resulted in amplicon size of ~ 500 bp (Fig. 4b and Suppl Fig. 1b) and CAL2-28F/CAL2-RD that resulted in amplification of 600 bp (Fig. 4c). The PCR amplicons of primer targeting regions of ITS1-5.8S-ITS2and TEF were sequenced, and the pathogen identified was *F. clamydosporum.* BLAST was used to find regions of similarity between the query sequence and the NCBI database sequence. The sequence of ITS with accession numbers MK503837.1, OM319485, OM319486, and sequence of TEF with accession number OM441207, and OM441208 were successfully published in GenBank (https://www.ncbi.nlm.nih.gov Tables 5 and 6). The PCR amplicons of primer CAL2-28F/CAL2-RD were sequenced, and the pathogen identified was confirmed as *F. clamydosporum.* Sequence of CAL2-28F/CAL2-RD with accession number OP345814, OP345815 were successfully published in GenBank (Table 7) The *F. clamydosporum* sequence was compared with its similar sequence available in the NCBI database. An optimal dendrogram was constructed using MEGA software v 5.05, and different taxa were clustered together in a bootstrap test 1000 replicates using sequences of ITS, TEF and CAL2 genes that were compared with their respective hits retrieved from NCBI database and compared with the already available* F. chlamydosporum* sequences (Fig 5). All the *Fusarium chlamydosporum* isolates obtained showed grouping with already known *Fusarium chlamydosporum*^19^.

**Table 5:** Accession number of isolates collected from different places (ITS).

S.No	Isolates	Place of collection	Pathogen identified	Host plant	Accession number (ITS)
1	A8	Srinagar-HMT	*Fusarium chlamydosporum*	Chili	MK503837.1
2	R	Baramulla-Sopore	*Fusarium chlamydosporum*	Chili	OM319485
3	S	Anantnag-Kokarnag	*Fusarium chlamydosporum*	Brinjal	OM319486

**Table 6:** Accession number of isolates collected from different places (TEF).

S. No	Isolates	Place of collection	Pathogen identified	host plant	Accession number (TEF)
1	R	Baramulla-Sopore	*Fusarium chlamydosporum*	Chili	OM441207
2	S	Anantnag-Kokarnag	*Fusarium chlamydosporum*	Brinjal	OM441208

**Table 7:** Accession number of isolates collected from different places (CAL2).

S. No	Isolates	Place of collection	Pathogen identified	Host plant	Accession number (CAL2)
1	R	Baramulla-Sopore	*Fusarium chlamydosporum*	Chili	OP345814
2	S	Anantnag-Kokarnag	*Fusarium chlamydosporum*	Brinjal	OP345815

## Discussion

Chili and brinjal are the most widely grown solanaceous crops worldwide and are rich source of nutrients, thus helping combat various human health diseases and disorders^1^. However, the production of these crops has drastically reduced from the last several years due to several biotic and abiotic stresses. The main constrain in producing these crops is the attack of various fungal diseases^3^.The current study was carried out to identify the causal pathogen of the most devastating wilt disease of chili and brinjal, to which 50–80% losses have been attributed. Previous reports have found that wilt in solanaceous crops is caused by *F. oxysporum*, *F. solani* and *F. equiseti*^5^. The pathogen isolates were collected from three districts (Srinagar, Baramulla, and Anantnag) in Kashmir Division, and were isolated and maintained on PDA and SNA media at 25 ± 1 ℃^20,21^ to study morpho-cultural characteristics. In this study, a new species of *Fusarium* causing wilt was identified based on morpho-cultural characteristics and confirmed by DNA Barcoding of ITS, TEF and Calmodulin genomic regions, identified as *Fusarium chlamydosporum.* Many researchers have utilized ITS and TEF genomic regions for identification of *Fusarium* species and have confirmed these primers as barcode DNA for identification of various species of fungi^21–24^ but for confirmation of the species identified CAL2-28F/CAL2-RD were also utilized because many researchers have earlier authenticated these primers for species identification in *Fusarium* pathogen^19,25^. Although *Fusarium chlamydosporum* was known to cause wilt in cotton, damping-off disease on Aleppo pine in Algeria, wilt disease of guava (*Psidium guajava* L.) in India, Die-back of olive in Tunisia, crown rot in Banana and lentil^26–29^ but first time is reported as causal agent of wilt disease in *Capsicum annuum* L. and *Solanum melongena* L.in Kashmir valley in Northern Himalayan region of India.

The inoculation of the cultured wilt pathogen on respective hosts using rhizosphere inoculation technique produced characteristic symptoms of wilt on leaves and vascular bundles with incubation periods of 19–20 days in *F. chlamydosporum,* in chili and brinjal hosts indicating same virulent nature of *F. chlamydosporum* in both host crops. The results are in complete agreement with other researchers who have reported almost same incubation period in different species of *Fusarium* infecting chili^5^.

Internal transcribed spacer (ITS) amplification using genus and/or species-specific ITS primers distinguished *F.chlamydosporum* based on PCR amplification using ITS primer combinations K-Lab-FusOxy-ITS1F2 and K-Lab-FusOxy-ITS4R2 on different isolates that showed amplification of ~ 500 bp. To further confirm our results, transcription elongation factor (TEF) amplification was carried out with TEF primer combination viz., K-Lab-TEF-Fu3-F and K-Lab-TEF-Fu3-R. Primer directed the amplification of ~ 500 bp. For reconfirming the identification CAL2-28F/CAL2-RD primers were used that showed amplification of 600 bp and the PCR amplicons were sequenced to reconfirm the species identified. The pathogens were identified using sequencing of the ITS1-5.8S-ITS2 and TEF regions and CAL2-28F/CAL2-RD (Tables 5, 6 and 7). Alignment of sequences through CLUSTALW revealed that the similarity among sequences was independent of their geographic origin, and it also showed the genetic relatedness of 45–98%.

In the phylogenetic study, *F.chlamydosporum* sequences of ITS, TEF and CAL2 genes were compared with their respective hits retrieved from NCBI database and were also compared with the already available *F.chlamydosporum* sequences^19^. The phylogenetic analysis led to the grouping of *F.clamydosporum* species sequences and their respective hits sequences and already available *F.chlamydosporum* sequences obtained from NCBI into different clades (Fig. 4c). Similar species were grouped in a single clade irrespective of their geographic origin. A new fungal species *F.chlamydosporum* was found associated with causing wilt of chili and brinjal from Kashmir valley, and this is the first report of this pathogen from India.

## Supplementary Information

Supplementary Table S1.

Supplementary Information.

## Data Availability

The sequencing data is available on NCBI database. The sequence of ITS with accession numbers MK503837.1, OM319485, OM319486, were successfully published in GenBank (https://www.ncbi.nlm.nih.gov). The sequence of TEFs have been provided Accession Numbers OM441207, and OM441208 by https://www.ncbi.nlm.nih.gov. The sequence of CAL2 have been provided Accession Numbers OP345814, OP34581 in GeneBank (https://www.ncbi.nlm.nih.gov.) Morphological identification was done by registered plant pathologist Dr. Z.A. Bhat. Molecular identification was done by Dr. Khalid Z. Masoodi. Voucher specimen have been deposited at K-Lab pathogen repository, Division of Plant Biotechnology, SKUAST-K, Shalimar. The experimental research and field studies carried out in the present investigation and plant collections are in accordance with local legislation and comply with institutional, national, and international guidelines.
