# Supplementary material for: Fusarium chlamydosporum, causing wilt disease of chili (Capsicum annum L.) and brinjal (Solanum melongena L.) in Northern Himalayas: a first report
Source: Sci Rep. 2022 Nov 27;12:20392. doi: 10.1038/s41598-022-23259-w (PMC9701669; doi:10.1038/s41598-022-23259-w)
Supplement: Supplementary file 1 — Supplementary Table S1. [file 41598_2022_23259_MOESM1_ESM.docx]

| Substrate | Quantity used |
| --- | --- |
| Sand | 90g |
| Maize meal agar/Corn meal agar (medium) | 10g |
| Distilled Water | 40ml |
| Spore suspension | 100µl/pot |

Supplementary Table 1: Preparation of potting mixture and inoculum for Pathogenicity test
