# Supplementary material for: Fusarium chlamydosporum, causing wilt disease of chili (Capsicum annum L.) and brinjal (Solanum melongena L.) in Northern Himalayas: a first report
Source: Sci Rep. 2022 Nov 27;12:20392. doi: 10.1038/s41598-022-23259-w (PMC9701669; doi:10.1038/s41598-022-23259-w)
Supplement: Supplementary file 2 — Supplementary Information. [file 41598_2022_23259_MOESM2_ESM.pptx]

## Slide 1
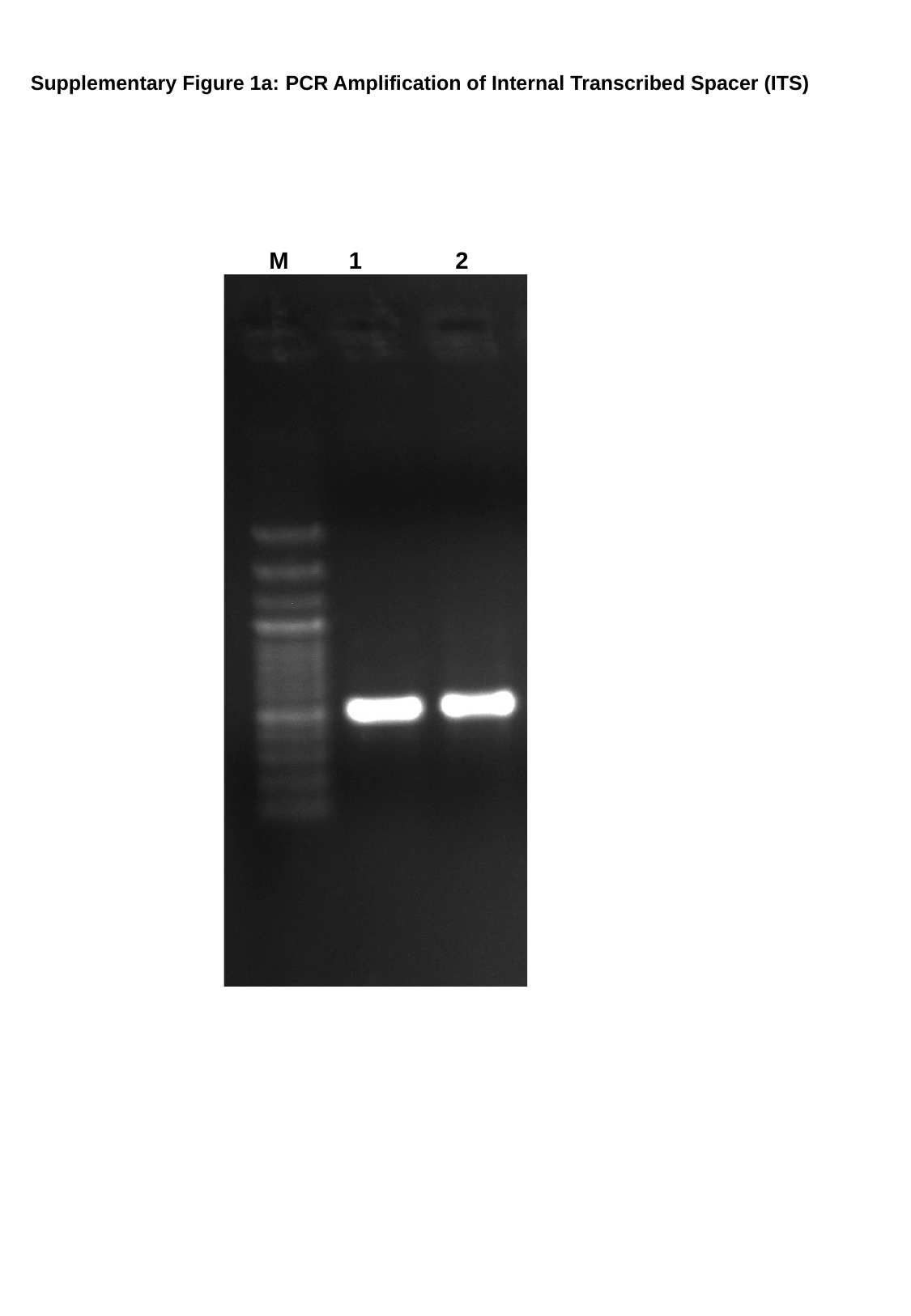

Supplementary Figure 1a: PCR Amplification of Internal Transcribed Spacer (ITS)
 M 1 2

## Slide 2
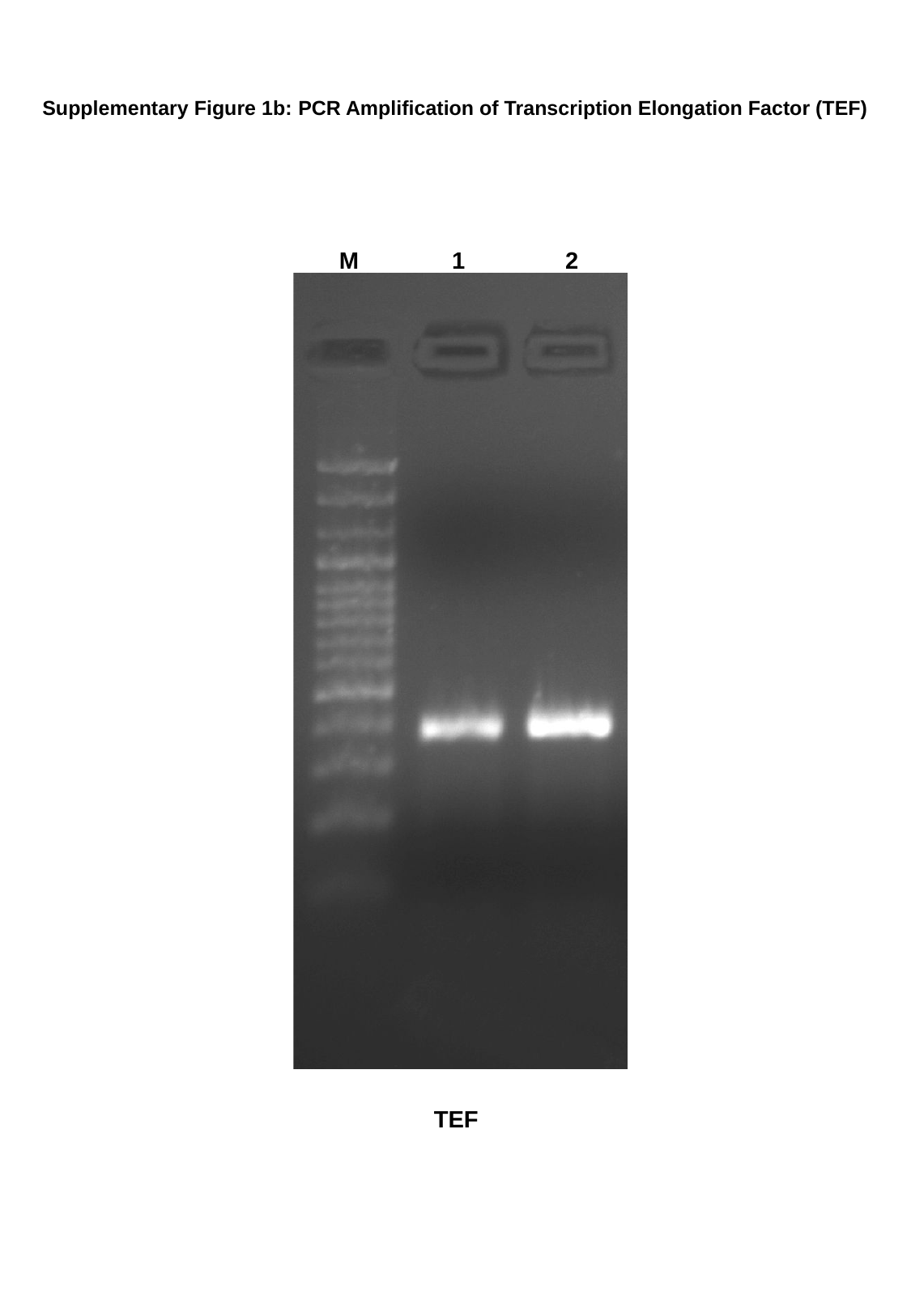

Supplementary Figure 1b: PCR Amplification of Transcription Elongation Factor (TEF)
 M 1 2
TEF

## Slide 3
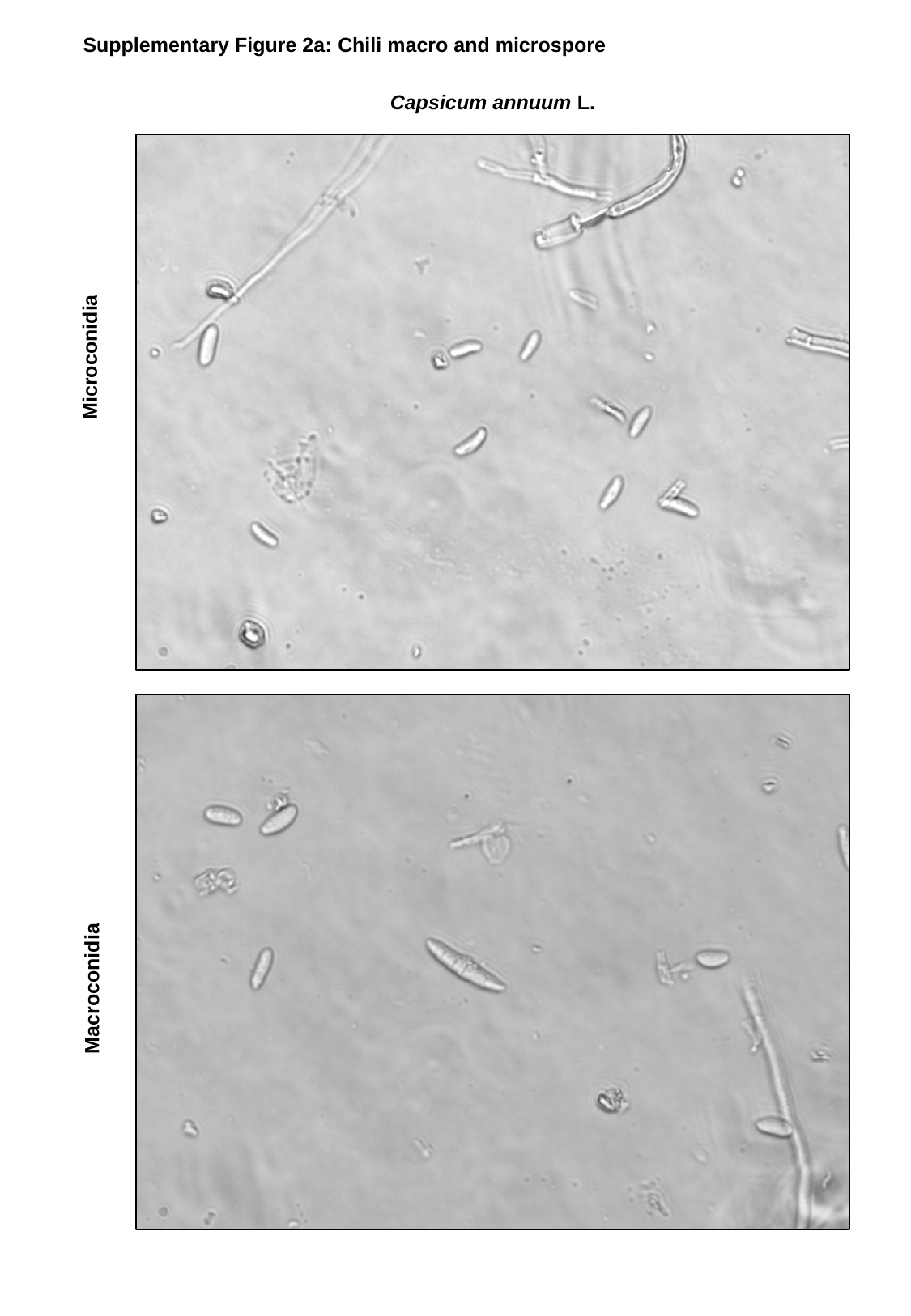

Supplementary Figure 2a: Chili macro and microspore
Capsicum annuum L.
Microconidia
Macroconidia
Macroconidia

## Slide 4
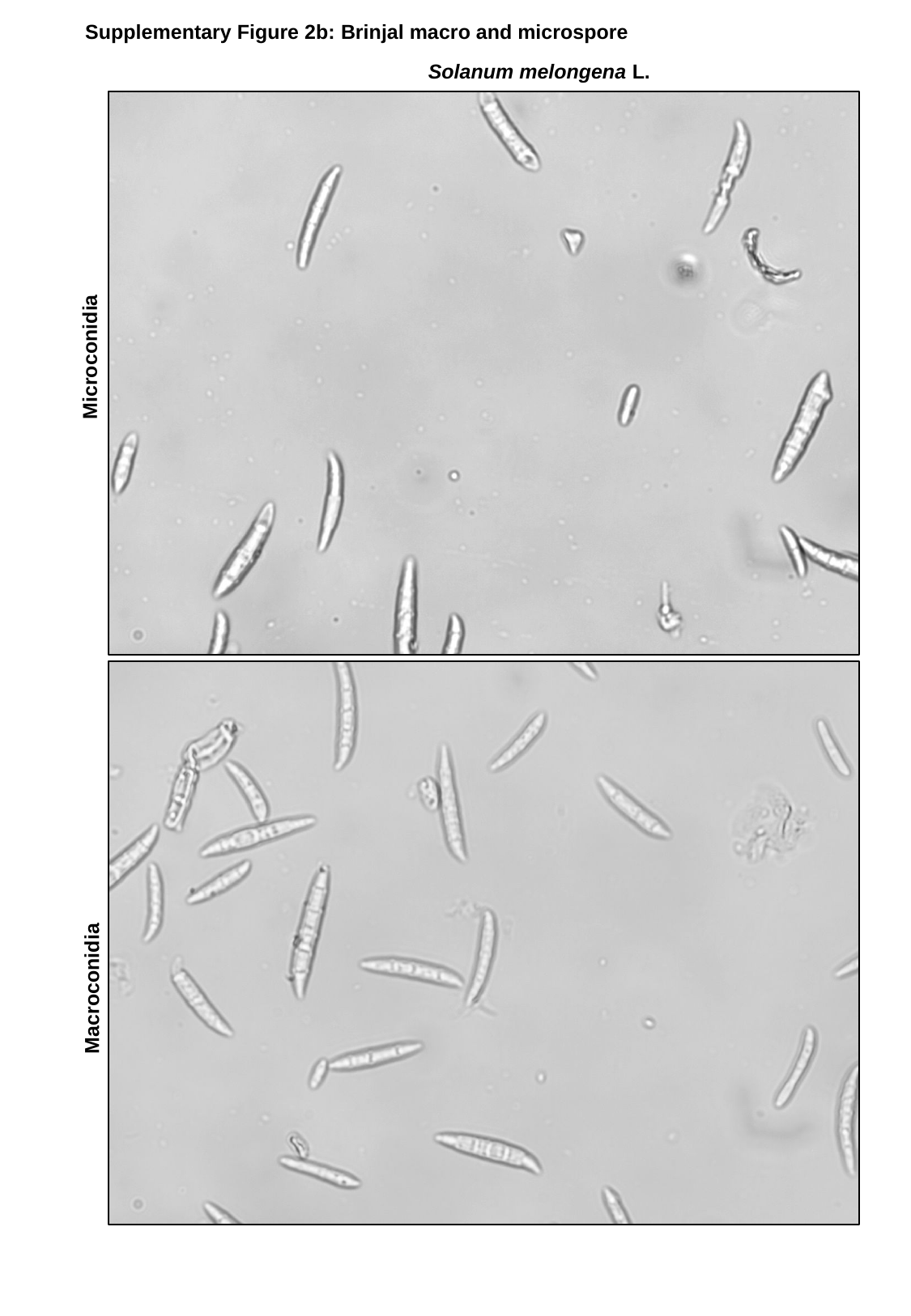

Supplementary Figure 2b: Brinjal macro and microspore
Solanum melongena L.
Microconidia
Macroconidia

## Slide 5
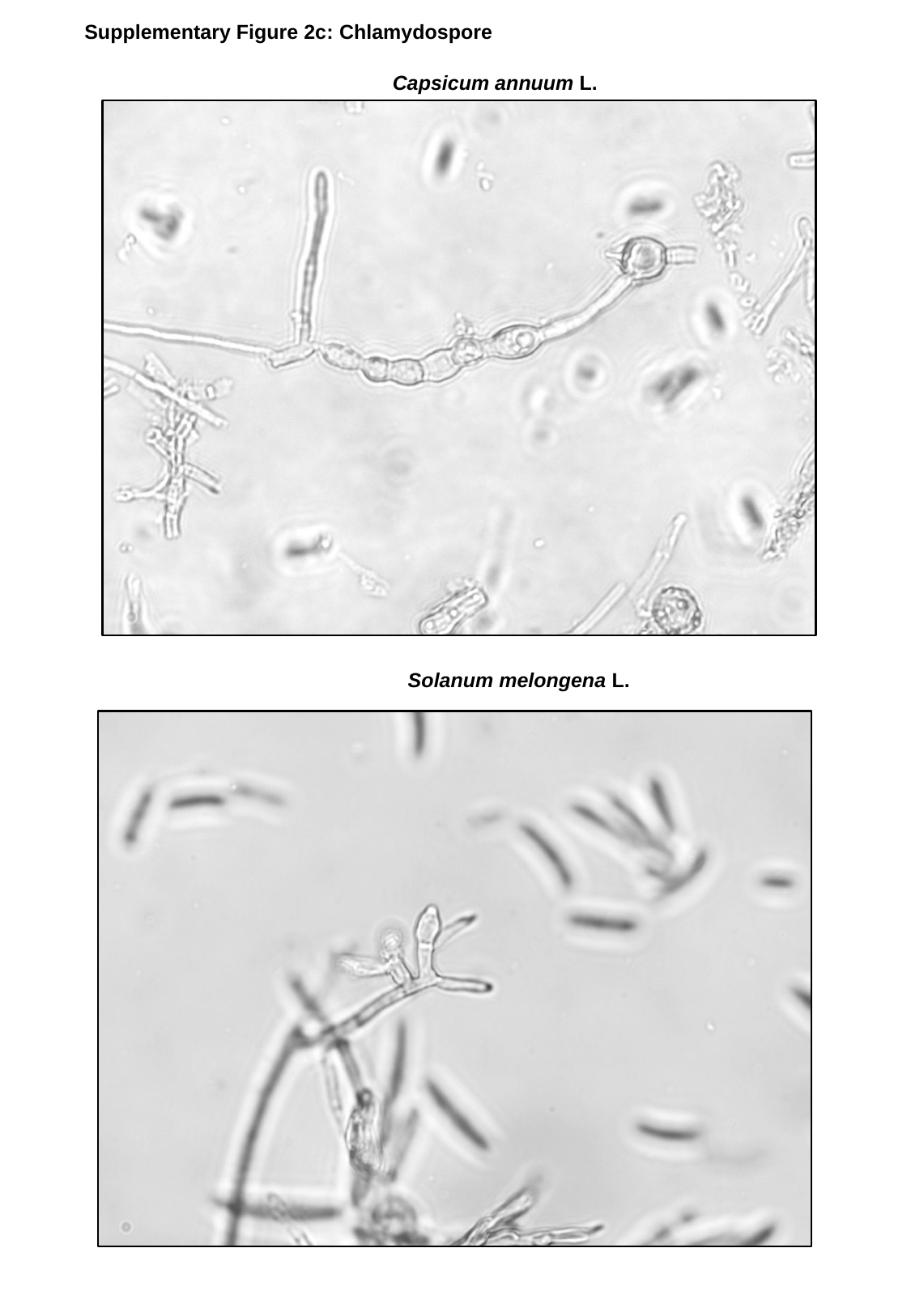

Supplementary Figure 2c: Chlamydospore
Capsicum annuum L.
Solanum melongena L.

## Slide 6
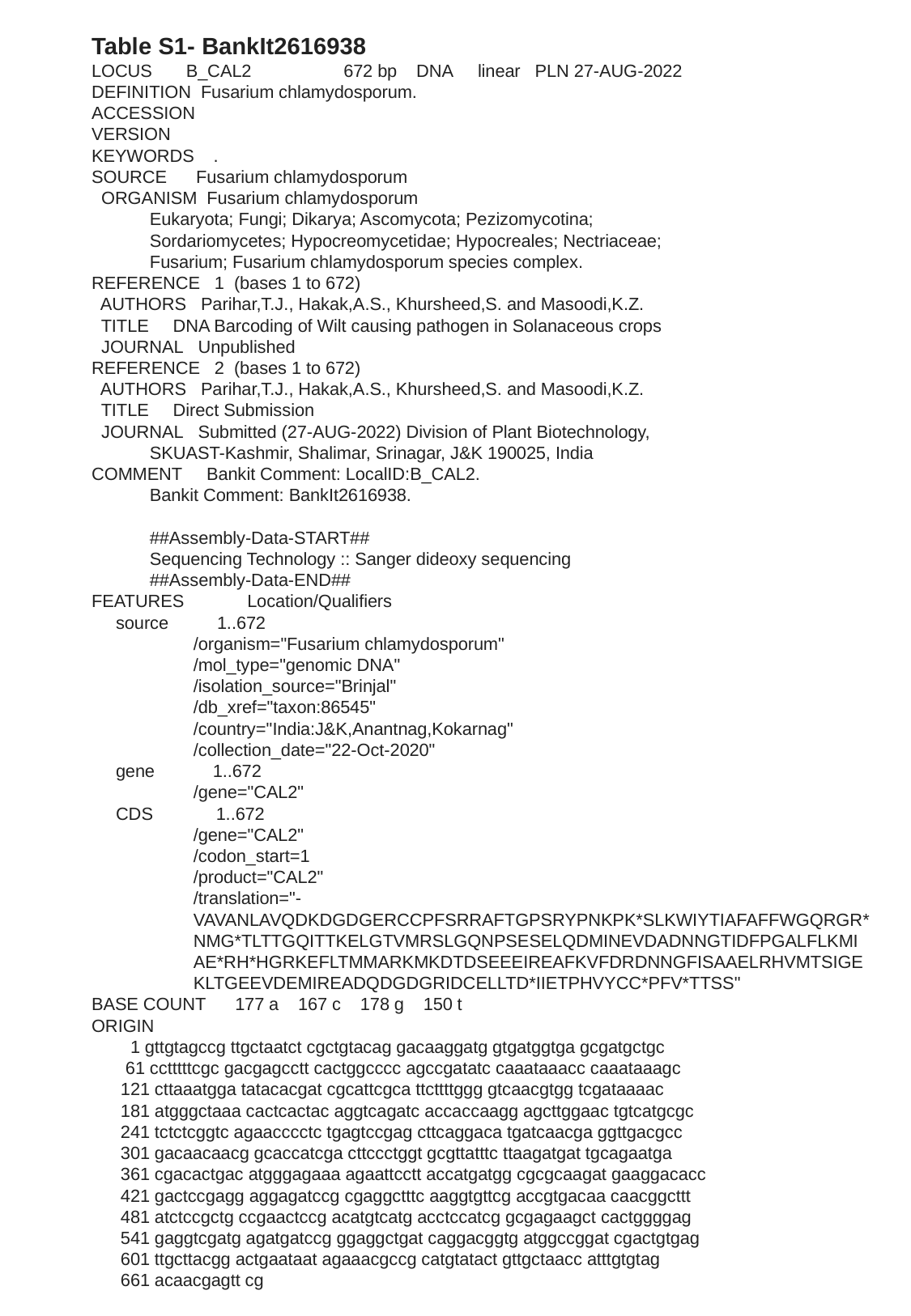

Table S1- BankIt2616938
LOCUS       B_CAL2                   672 bp    DNA     linear   PLN 27-AUG-2022
DEFINITION  Fusarium chlamydosporum.
ACCESSION   
VERSION
KEYWORDS    .
SOURCE      Fusarium chlamydosporum
  ORGANISM  Fusarium chlamydosporum
            Eukaryota; Fungi; Dikarya; Ascomycota; Pezizomycotina;
            Sordariomycetes; Hypocreomycetidae; Hypocreales; Nectriaceae;
            Fusarium; Fusarium chlamydosporum species complex.
REFERENCE   1  (bases 1 to 672)
  AUTHORS   Parihar,T.J., Hakak,A.S., Khursheed,S. and Masoodi,K.Z.
  TITLE     DNA Barcoding of Wilt causing pathogen in Solanaceous crops
  JOURNAL   Unpublished
REFERENCE   2  (bases 1 to 672)
  AUTHORS   Parihar,T.J., Hakak,A.S., Khursheed,S. and Masoodi,K.Z.
  TITLE     Direct Submission
  JOURNAL   Submitted (27-AUG-2022) Division of Plant Biotechnology,
            SKUAST-Kashmir, Shalimar, Srinagar, J&K 190025, India
COMMENT     Bankit Comment: LocalID:B_CAL2.
            Bankit Comment: BankIt2616938.
            ##Assembly-Data-START##
            Sequencing Technology :: Sanger dideoxy sequencing
            ##Assembly-Data-END##
FEATURES             Location/Qualifiers
     source          1..672
                     /organism="Fusarium chlamydosporum"
                     /mol_type="genomic DNA"
                     /isolation_source="Brinjal"
                     /db_xref="taxon:86545"
                     /country="India:J&K,Anantnag,Kokarnag"
                     /collection_date="22-Oct-2020"
     gene            1..672
                     /gene="CAL2"
     CDS             1..672
                     /gene="CAL2"
                     /codon_start=1
                     /product="CAL2"
                     /translation="-
                     VAVANLAVQDKDGDGERCCPFSRRAFTGPSRYPNKPK*SLKWIYTIAFAFFWGQRGR*
                     NMG*TLTTGQITTKELGTVMRSLGQNPSESELQDMINEVDADNNGTIDFPGALFLKMI
                     AE*RH*HGRKEFLTMMARKMKDTDSEEEIREAFKVFDRDNNGFISAAELRHVMTSIGE
                     KLTGEEVDEMIREADQDGDGRIDCELLTD*IIETPHVYCC*PFV*TTSS"
BASE COUNT      177 a    167 c    178 g    150 t
ORIGIN     
        1 gttgtagccg ttgctaatct cgctgtacag gacaaggatg gtgatggtga gcgatgctgc
       61 cctttttcgc gacgagcctt cactggcccc agccgatatc caaataaacc caaataaagc
      121 cttaaatgga tatacacgat cgcattcgca ttcttttggg gtcaacgtgg tcgataaaac
      181 atgggctaaa cactcactac aggtcagatc accaccaagg agcttggaac tgtcatgcgc
      241 tctctcggtc agaacccctc tgagtccgag cttcaggaca tgatcaacga ggttgacgcc
      301 gacaacaacg gcaccatcga cttccctggt gcgttatttc ttaagatgat tgcagaatga
      361 cgacactgac atgggagaaa agaattcctt accatgatgg cgcgcaagat gaaggacacc
      421 gactccgagg aggagatccg cgaggctttc aaggtgttcg accgtgacaa caacggcttt
      481 atctccgctg ccgaactccg acatgtcatg acctccatcg gcgagaagct cactggggag
      541 gaggtcgatg agatgatccg ggaggctgat caggacggtg atggccggat cgactgtgag
      601 ttgcttacgg actgaataat agaaacgccg catgtatact gttgctaacc atttgtgtag
      661 acaacgagtt cg

## Slide 7
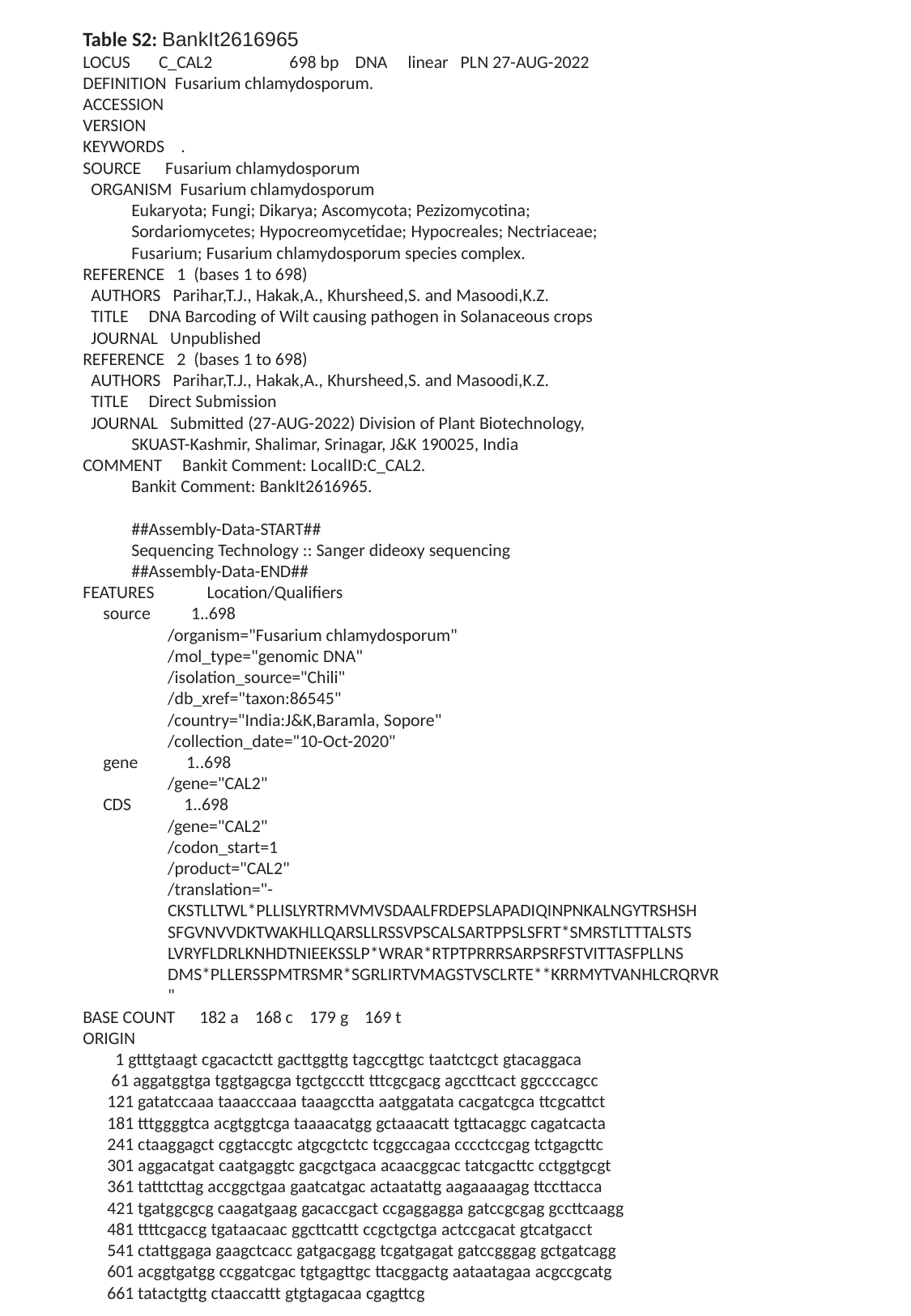

Table S2: BankIt2616965
LOCUS       C_CAL2                   698 bp    DNA     linear   PLN 27-AUG-2022
DEFINITION  Fusarium chlamydosporum.
ACCESSION   
VERSION
KEYWORDS    .
SOURCE      Fusarium chlamydosporum
  ORGANISM  Fusarium chlamydosporum
            Eukaryota; Fungi; Dikarya; Ascomycota; Pezizomycotina;
            Sordariomycetes; Hypocreomycetidae; Hypocreales; Nectriaceae;
            Fusarium; Fusarium chlamydosporum species complex.
REFERENCE   1  (bases 1 to 698)
  AUTHORS   Parihar,T.J., Hakak,A., Khursheed,S. and Masoodi,K.Z.
  TITLE     DNA Barcoding of Wilt causing pathogen in Solanaceous crops
  JOURNAL   Unpublished
REFERENCE   2  (bases 1 to 698)
  AUTHORS   Parihar,T.J., Hakak,A., Khursheed,S. and Masoodi,K.Z.
  TITLE     Direct Submission
  JOURNAL   Submitted (27-AUG-2022) Division of Plant Biotechnology,
            SKUAST-Kashmir, Shalimar, Srinagar, J&K 190025, India
COMMENT     Bankit Comment: LocalID:C_CAL2.
            Bankit Comment: BankIt2616965.
            ##Assembly-Data-START##
            Sequencing Technology :: Sanger dideoxy sequencing
            ##Assembly-Data-END##
FEATURES             Location/Qualifiers
     source          1..698
                     /organism="Fusarium chlamydosporum"
                     /mol_type="genomic DNA"
                     /isolation_source="Chili"
                     /db_xref="taxon:86545"
                     /country="India:J&K,Baramla, Sopore"
                     /collection_date="10-Oct-2020"
     gene            1..698
                     /gene="CAL2"
     CDS             1..698
                     /gene="CAL2"
                     /codon_start=1
                     /product="CAL2"
                     /translation="-
                     CKSTLLTWL*PLLISLYRTRMVMVSDAALFRDEPSLAPADIQINPNKALNGYTRSHSH
                     SFGVNVVDKTWAKHLLQARSLLRSSVPSCALSARTPPSLSFRT*SMRSTLTTTALSTS
                     LVRYFLDRLKNHDTNIEEKSSLP*WRAR*RTPTPRRRSARPSRFSTVITTASFPLLNS
                     DMS*PLLERSSPMTRSMR*SGRLIRTVMAGSTVSCLRTE**KRRMYTVANHLCRQRVR
                     "
BASE COUNT      182 a    168 c    179 g    169 t
ORIGIN     
        1 gtttgtaagt cgacactctt gacttggttg tagccgttgc taatctcgct gtacaggaca
       61 aggatggtga tggtgagcga tgctgccctt tttcgcgacg agccttcact ggccccagcc
      121 gatatccaaa taaacccaaa taaagcctta aatggatata cacgatcgca ttcgcattct
      181 tttggggtca acgtggtcga taaaacatgg gctaaacatt tgttacaggc cagatcacta
      241 ctaaggagct cggtaccgtc atgcgctctc tcggccagaa cccctccgag tctgagcttc
      301 aggacatgat caatgaggtc gacgctgaca acaacggcac tatcgacttc cctggtgcgt
      361 tatttcttag accggctgaa gaatcatgac actaatattg aagaaaagag ttccttacca
      421 tgatggcgcg caagatgaag gacaccgact ccgaggagga gatccgcgag gccttcaagg
      481 ttttcgaccg tgataacaac ggcttcattt ccgctgctga actccgacat gtcatgacct
      541 ctattggaga gaagctcacc gatgacgagg tcgatgagat gatccgggag gctgatcagg
      601 acggtgatgg ccggatcgac tgtgagttgc ttacggactg aataatagaa acgccgcatg
      661 tatactgttg ctaaccattt gtgtagacaa cgagttcg
